# The Contribution of Meaning Making and Religiosity to Individuals’ Psychological Wellbeing During the COVID-19 Pandemic: Prosocial Orientation Matters

**DOI:** 10.5964/ejop.9389

**Published:** 2023-05-31

**Authors:** Daniela Villani, Angela Sorgente, Alessandro Antonietti, Paola Iannello

**Affiliations:** 1 Department of Psychology, Università Cattolica del Sacro Cuore of Milan, Milan, Italy; 1 University of Bari Aldo Moro, Bari, Italy

**Keywords:** COVID-19 pandemic, flourishing, psychological well-being, religiosity, meaning making, emerging adults

## Abstract

The COVID-19 pandemic has dramatically affected individuals’ psychological well-being worldwide, thus representing a challenge for flourishing among emerging adults. To understand psychological processes involved in the positive adaptation to this challenge, the present study examined the role of meaning in life and religious identity as crucial resources for flourishing in a sample of 255 Italian emerging adults. Specifically, as in the midst of a stressful event individuals may experience the potential for flourishing through the process of search for meaning, the study examined the mediated role of existential, spiritual/religious and prosocial orientations as the three primary trajectories for building meaning. Results from path analytic mediation models revealed a positive influence of presence of meaning and in-depth exploration on flourishing. Findings also suggested the contribution of prosocial orientation in building meaning and, ultimately, in increasing flourishing. Implications are discussed.

In 2021 high rates of COVID-19 cases have been reported worldwide. Initial responses to contain the infection included non-pharmacological interventions, such as lockdowns, remote working and online schooling, and social distancing. These interventions, which have been unquestionably effective to slow down the spread of the virus and protect physical health (Nussbaumer-Streit et al., 2020), have required significant changes in individuals’ behavioral patterns that impacted on their mental health and well-being (Brooks et al., 2020; Hossain et al., 2020). Furthermore, the lack of personal control when facing uncertain, unpredictable and uncontrollable events, represents another critical source of threaten and stress (Evans et al., 2001).

Thus, a large number of studies highlighted the adverse impact of COVID-19 pandemic on subjective and psychological well-being across different ages and countries, including Italy (Favieri et al., 2021; Mazza et al., 2020; Pagnini et al., 2020; Paleari et al., 2021; Papapicco et al., 2021; Petrocchi et al., 2022).

## The Protective Role of Meaning Making and Religiosity

Meaning making and religiosity represent two important resources that could act as protective factors against the negative impact of the COVID-19 pandemic on people’s psychological well-being. Finding life meaningful is one of the inner processes that may enhance the perception of control over the situation and reduce the negative impact of stressors. The presence of meaning in life implies the belief that what happens in the world is coherent, predictable, and controllable (Baumeister & Vohs, 2002; Heintzelman & King, 2014). Hence, people who consider life as meaningful tend to perceive also a sense of control over adverse situations. Similarly, constructing meaningful interpretations of highly negative events has been found to be an effective response for dealing with such stressors, which facilitates adaptation processes (Silver & Updegraff, 2013). In dealing with COVID-19 emergency, meaning in life has been found to have a positive relationship with the ability to cope with the situation (Karataş & Tagay, 2021), confirming its protective role on mental health (Arslan et al., 2021; Trzebiński et al., 2020).

Another personal resource that may play an important role in coping with adverse situations is religiosity. In times of uncertainty, it is not surprising that people might turn to religion for solace. Indeed, there is some compelling evidence for a rise in religiosity in people who are facing negative and unpredictable situations, such as severe illness or deaths in close family members (Sinding Bentzen, 2019). Positive religious coping has been associated with various favorable outcomes, such as reduced depression, anxiety, and increased psychological well-being (Koenig, 2018).

For many people, religion as “a search for significance in ways related to the sacred” (Pargament, 1997, p. 32) represents a core schema that informs their beliefs about themselves and the world and provides understanding of both ordinary and extraordinary occurrences (Spilka et al., 2003). Religiosity is thus strongly and positively linked to a sense of meaning in life (Steger & Frazier, 2005) and may be a powerful resource for mental health since it involves a framework of meaning-making associated with decreased psychological distress and a value-based pursuit of psychological well-being (Rosmarin & Koenig, 2020; Steger, 2018).

## Rebuilding Meaning Through Re-Orientation Paths

The pandemic represents a challenge and according to several recent studies (Hooker et al., 2020; Trzebiński et al., 2020) cultivating meaning in the midst of this ongoing pandemic represents a protective factor for mental health and well-being. By focusing on the development of restoring and rebuilding meaning in the midst of a distressing experience, people may experience the potential for growth and flourishing (Van Tongeren & Van Tongeren, 2021), intended as the optimal psychological functioning accompanied by feelings of meaning, engagement, and purpose in life (Keyes, 2002; Ryan & Deci, 2001).

The cultivation of meaning is an active process that allows people to metabolize experiences of suffering in ways that can lead to transformative expressions of the good life. To investigate this process of reflection and re-orientation, we draw from three relevant areas of empirical research that share a common feature of meaning (Van Tongeren & Van Tongeren, 2021). Specifically, we focus on existential issues (i.e., questioning about the origin and finality of the world and the meaning of life), spirituality and religiosity (i.e., transcending the self and connecting with the divine), and prosociality (i.e., improving others’ lives) as three primary trajectories for building meaning that can lead to flourishing.

First, the exploration of existential issues represents a valuable dimension in the promotion of psychological well-being, which sustains the realization of human strengths and virtues (Ryan & Deci, 2001). Addressing the fundamental questions of existence is a universal human experience that crosses cultures and religious status and may be important for optimal individual functioning (Ryff, 2014).

Second, when people consider their global meanings about life and death, they often refer to the sacred aspect involved in both the definition of religiousness and spirituality (Zinnbauer & Pargament, 2005). The sacred includes concepts such as the divine, God, and the transcendent dimension, which provide an ultimate meaning to life and a sense of personal security and safety toward the unknown (Pargament et al., 2005). When people build meaning through connections to religion and spirituality (Park, 2013), they address the stress of existential concerns and transcend themselves to live more flourishing lives (Myers, 2008). For some people, religiosity can represent a key aspect of identity that people rely upon to cope with adversity (Aten et al., 2019) and may facilitate coping with uncontrollable situations, such as the COVID-19 pandemic, especially through an intensification of prayer activity, intended as a way of dealing with hardship and seeking closeness to God (Pirutinsky et al., 2020; Sinding Bentzen, 2020). But not everyone focuses on a religion-oriented spirituality; nontheistic seekers may still try to look beyond the current situation and physical limitations experienced in order to transcend their own boundaries, to pursue and engage with a higher purpose, mission, or vocation connected to or serving a greater good (Gan et al., 2023).

Finally, relationships constitute a primary source of meaning in life and research showed that the active participation to others’ well-being is associated with greater meaning in life (Van Tongeren et al., 2016) and that focusing on others, compared to focusing on oneself, enhances psychological flourishing (Nelson & Padilla-Walker, 2013). With the umbrella term of prosociality, we refer to prosocial and altruistic behaviors that share common aspects: they both reflect acts of positive social behavior towards one or several others, namely acts that are often being intended to promote the welfare of others (Penner et al., 2005; Pfattheicher et al., 2022).

## The Present Study

Many studies found that the pandemic has strongly decreased emerging adults’ well-being and mental health (Germani et al., 2020; Liu et al., 2020). Emerging adults are individual aged 18–30 years (Arnett, 2014), who are no more adolescent, but not yet adult: their adulthood is still “emerging” as, during the third decade of life, they have to accomplish some transitions to reach adulthood markers (Billari & Liefbroer, 2010). Since emerging adults are called to face complex and uncertain challenges, it becomes particularly relevant to identify those factors that might contribute to emerging adults flourishing during the COVID-19 era.

To better understand psychological processes that may sustain emerging adults’ flourishing in facing the COVID-19 pandemic, the present study examined the role of meaning in life and religious identity as principal resources (Gutierrez & Park, 2015). Furthermore, we considered existential, spiritual/religious, and prosocial orientations as the three primary trajectories for building meaning that can lead to flourishing.

Given the individuals’ differences in religiosity, we distinguished people strongly committed to religion (believers), people with weak or unsure belief (uncertain), and people with complete non-belief (completely non-religious or atheists), without collapsing the non-religious individuals and the uncertain ones (Galen & Kloet, 2011; Villani et al., 2019).

Specifically, the present study aims at: (1) investigating the role of meaning in life on emerging adults’ flourishing during pandemic both directly and indirectly through the orientations processes. We also tested whether the relationships in the model were invariant across the three different religious statuses (believers, atheists, and uncertain); (2) examining the role of religious identity on emerging adults’ flourishing during pandemic both directly and indirectly through the orientations processes. We also tested whether the relationships in the model were invariant across the two groups who have some sort of religious beliefs (believers and uncertain).

## Method

### Participants

The convenient sample was composed by 255 Italian emerging adults (87.5% female) aged 18–30 (*M* = 21.64; *SD* = 2.37). Most participants had high school diploma (69.8%) or degree (29.0%) as highest level of education. Regarding their religious status, most of the participants reported to be believers (44.2%) or to have unsure beliefs about their religious status (44.3%; i.e., they stated to be neither religious nor non-religious). The remaining 14.5% declared to be atheist. Religious and uncertain participants were also asked to indicate which religion they belong to and 92.5% of them reported to be Christian (mainly Catholic). Few respondents selected other options, like Islamic (1.5%) or Buddhist (1.5%).

### Procedure

An advertisement for research participation containing a hyperlink to a survey was first sent by email to authors’ personal contacts. Then, the sample was recruited through the method of non-random snowball sampling. The only inclusion criterion concerned the emerging adulthood age range (18–30; Arnett, 2014).

Participants volunteered to respond to the online survey, which took approximately 20 minutes to complete. Each of them signed an online informed consent. Considering the anonymous, voluntary and minimal risk associated with participating in this online survey where no personal data were provided, ethical approval from a research ethics committee (REC) was considered not to be an absolute requirement. All procedures performed in the study involving human participants were in accordance with the ethical standards of the Declaration of Helsinki.

### Measures

The online survey included the following measurement scales:

#### Meaning in Life

The perceived meaning in life was assessed through the Italian validation (Negri et al., 2020) of the 10-item Meaning in Life Questionnaire (Steger et al., 2006), measuring the *Presence* of meaning in life, referring to perceived meaning and purpose in life (e.g., “My life has a clear sense of purpose”) and the *Search* for meaning in life, referring to the active commitment to find meaning in life (e.g., “I am always looking to find my life’s purpose”) (α = .854 and α = .871 respectively) through a 7-point scale from 1 (absolutely untrue) to 7 (absolutely true).

#### Religious Identity

Within the broader construct of religiousness (or religiosity) concerning the public or private adherence to beliefs and rituals of a religion, here we focused on religious identity conceived as the extent to which people self-identify with a faith tradition/community (Lopez et al., 2011). The religious identity formation was measured by the 13-item Utrecht-Management of Identity Commitments Scale (U-MICS; Crocetti et al., 2008) recently validated in Italian language within the religious domain (Sorgente et al., 2022). The scale is composed of three subscales, each corresponding to a different identity formation process: the 5-item *Commitment* subscale indicates the process of strong engagement in a religion; The 5-item *In-Depth Exploration* subscale refers to the process of active probing own current religious commitment; The 3-item *Reconsideration of Commitment* subscale pertains to the process of questioning different religious commitment when current religion is no longer fulfilling. Items were rated on a 5-point scale from 1 (completely untrue) to 5 (completely true). As items referred to “my religion”, we prefer not to administer this scale to atheists, in accordance with previous publications (Villani et al., 2019). All the subscales were highly reliable in our sample, respectively α = .949, α = .870, and α = .849.

#### Orientation Processes

The orientation processes that people activated due to their COVID-19 experience was measured through an *ad hoc* questionnaire. No pre-existing questionnaire was available, thus the questionnaire was designed by this research group in accordance with the literature (Van Tongeren & Van Tongeren, 2021). The *ad hoc* questionnaire aims at measuring three distinct processes that individual may rely on to cope with the COVID-19 experience, namely reflecting on existential concerns (“existential orientation”), cultivating spirituality and religiosity (“spiritual/religious orientation”), and focusing on others (“prosocial orientation”). The questionnaire includes eight items rated on a 5-point scale (1 = completely untrue; 5 = completely true) concerning each specific orientation path that the COVID-19 diffusion could have activated in participants (“The COVID-19 pandemic outbreak...”). In particular, we developed two items measuring the existential orientation (e.g., “led me to reflect on issues such as the fragility of existence, the meaning of life, the destiny of humanity”), three items measuring the spiritual/religious orientation (e.g., “induced me to read texts—papers, books, Internet sites, etc.—on spiritual and/or religious issues”), and three items measuring the prosocial orientation (e.g., “has increased my awareness and sensitivity to other people’s problems and social issues”). After verifying that the expected factorial structure was confirmed [χ^2^ (17) = 36.284; *p* = .0042; RMSEA = 0.067 (0.036 0.097); *p* = 0.518; CFI = 0.951], we estimated the reliability of each subscale: α = .669, .733, and .704. See Table S1 and Table S2 in the Supplementary Materials section for more details.

#### Flourishing

Emerging adults’ psychological well-being was assessed using the Italian validation (Giuntoli et al., 2017) of the Flourishing Scale (Diener et al., 2010). This mono-dimensional scale includes eight items assessed on a 7-point scale (1 = strongly disagree; 7 = strongly agree) and was highly reliable in our sample (α = .818).

#### Covid-19 Impact

We administered eight items developed *ad hoc* to rate on a 5-point scale, from (1 = not at all) to (5 = a lot), how much the COVID-19 have impacted participants’ life domains (i.e., personal/relatives’ health, family relationships, personal/family finances, work activities, personal course of study). At the time of this writing no appropriate validated pre-existing tool was available, thus the research team specifically constructed the eight items of the questionnaire in accordance with the literature, which identified specific key life domains (see Ryan et al., 2021). After confirming that this scale was mono-dimensional, χ^2^(20) = 52.388, *p* = < .001; RMSEA = 0.080 , 95% CI [0.054, 0.107], *p* = 0.031; CFI = 0.915, and reliable (α = .772), we adopted it as control variable in our statistical models.

### Data Analysis

Firstly, we verified if the mean level of the measured variables was different across the three groups here investigated (believers, uncertain, and atheists) by running, for each variable, a one-way ANOVA though SPSS and using the Fisher's Least Significant Difference (LSD) test for post-hoc comparisons. Secondly, we performed two path analytic mediation models, one for each predictor (meaning in life and religious identity) in order to verify their total, direct, and indirect (via the orientation processes) impact on participants’ flourishing. These models were run in Mplus adopting the Maximum Likelihood as estimation method and the Full Information Maximum Likelihood as method to manage missing data.

#### Meaning in Life Model

The model testing the meaning in life as predictor was run on the entire sample (*n* = 252). To address our mediation hypothesis, we constructed a saturated path analytic mediation model and estimated direct, indirect, and total effects linking the two meaning in life dimensions (presence and search for meaning in life) to emerging adults’ flourishing, directly and indirectly via the three parallelly mediating orientation processes (existential, spiritual/religious, and prosocial orientation). Bootstrapping procedure was used to estimate bias-corrected confidence intervals (95% CIs based on 10,000 randomly sampled subsets; Stride et al., 2015) for indirect effects.

To account for the effect of COVID-19 impact on this mediating process, we include the COVID-19 impact score as a control variable in this model. Finally, using the multiple group analysis approach and parameter constraints, we tested if parameters in our model were equivalent (i.e., structural invariance) across the three religious status groups (believers, uncertain, and atheists). Parameters were considered equivalent if the constrained model was not significantly different (i.e., Δχ^2^ with a *p* > .05; Bollen, 1989) from the freed model.

#### Religious Identity Model

The model testing the religious identity as predictor was run only on believer and uncertain emerging adults (*n* = 216), as the religious identity scale was not administered to atheists. In this case, the path analytic mediation model estimated direct, indirect, and total effects linking the three religious identity dimensions (commitment, in-depth exploration, and reconsideration of the commitment) to emerging adults’ flourishing, directly and indirectly via the three mediating orientation processes.

As for the previous model, the indirect effects’ confidence intervals were estimated using bootstrapping procedure and the COVID-19 impact score was included as a control variable in the model. The only difference from the previous model is that the structural invariance of the model was tested across two groups (believers and uncertain) instead of three groups.

## Results

### Descriptive Statistics

In Table 1 we reported mean and standard deviation for each variable separately for diverse participants’ religious statuses and verified whether the means were significantly different across groups.

**Table 1:** Descriptive Statistics, M (SD), and Mean Comparisons For All Study Variables

Psychological dimension	Believer	Uncertain	Atheist	ANOVA result
Presence of meaning	4.85 (1.03)_a_	4.54 (1.24)_b_	4.38 (1.25)_b_	*F*(2, 252) = 3.16; *p* = .044; η^2^ = .024
Search for meaning	5.20 (1.25)	5.33 (1.07)	4.93 (1.46)	*F(*2, 252) = 2.20; *p* = .227; η^2^ = .012
Commitment	3.25 (0.83)	2.05 (0.88)	/	*F*(1, 214) = 105.93; *p* < .001; η^2^ = .331
In-depth exploration	3.31 (0.77)	2.41 (0.97)	/	*F*(1, 214) = 56.46; *p* < .001; η^2^ = .209
Reconsideration	1.61 (0.77)	1.97 (0.85)	/	*F*(1, 214) = 10.92; *p* = .001; η^2^ = .049
Existential orientation	3.61 (0.80)_a_	3.32 (0.96)_b_	2.86 (0.98)_c_	*F*(2, 249) = 10.25; *p* < .001; η^2^ = .076
Spiritual/religious orientation	2.32 (0.82)_a_	1.81 (0.80)_b_	1.42 (0.73)_c_	*F*(2, 248) = 21.14; *p* < .001; η^2^ = .146
Prosocial orientation	3.66 (0.79)_a_	3.42 (0.83)_b_	3.26 (0.90)_b_	*F*(2, 248) = 4.20; *p* = .016; η^2^ = .033
Flourishing	5.70 (0.66)_a_	5.47 (0.81)_b_	5.40 (0.88)_b_	*F*(2, 249) = 3.49; *p* = .032; η^2^ = .027
COVID-19 impact	2.80 (0.86)	2.84 (0.79)	2.64 (0.76)	*F*(2, 249) = 0.82; *p* = .441; η^2^ = .007

*Note*. Statistics about religious identity dimensions are not available for atheists because the instrument measuring religious identity was not administered to them. A mean is significantly different from another mean if they have different subscripts (i.e., a, b, c). A mean without a subscript is not significantly different from any other mean.

Results indicated that the means of search for meaning and COVID-19 impact were not significantly different across groups. Regarding the three dimensions of the religious identity, commitment and in-depth exploration were significantly higher in believers than uncertain emerging adults, while the reconsideration of commitment was higher for those with uncertain beliefs. Regarding the orientation factors, both the existential and the spiritual/religious ones were higher in believers than uncertain emerging adults, who, in turn, had higher levels than atheists. Prosocial orientation was higher for believers than uncertain and atheists. Finally, presence of meaning and flourishing were both higher for believers than uncertain and atheist emerging adults.

### Meaning in Life Model

**Figure 1:**
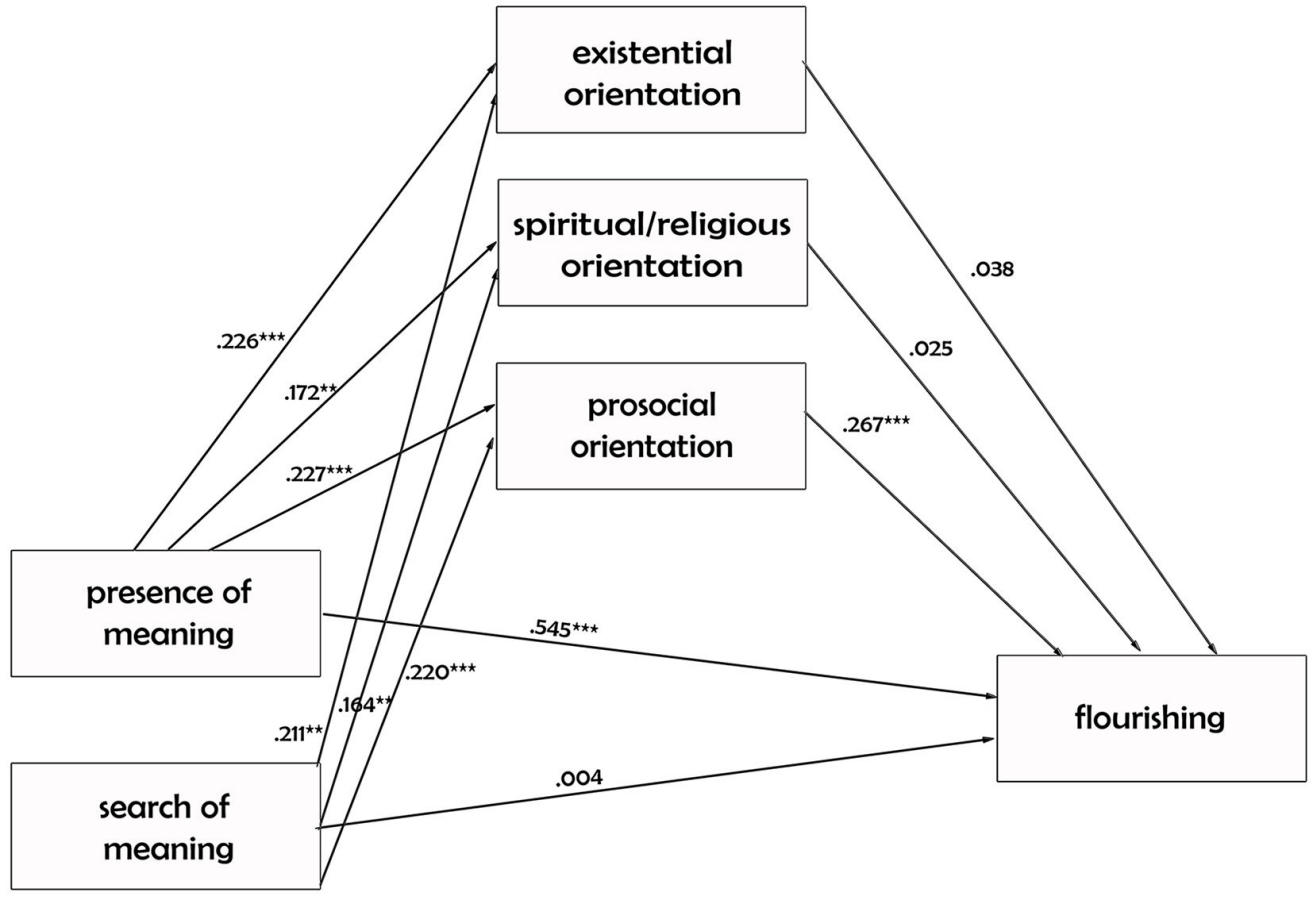
The Direct and Indirect Influence of Meaning in Life on Flourishing *Note.* Saturated path analytic mediation model testing the direct and indirect influence of meaning in life on flourishing (*n* = 252). For the sake of simplicity, correlation between the two predictors as well as correlations among the three mediators are not represented. Standardized values are reported. *p < .05. **p < .01. ***p < .001.

The first model testing the relationship between meaning in life’s dimensions and psychological well-being, via three mediators (existential, spiritual/religious, and prosocial orientation) was tested on the entire sample (Figure 1).

While in the figure only the direct effects are represented, in Table 2 we reported the direct, indirect, and total effects tested in this first model. In particular, we found that the presence of meaning in life has a strong total effect (β = .619) on flourishing. This total effect is divisible in two effects: a direct positive effect (β = .545) that the subjective sense that one’s life is meaningful has on the flourishing and an indirect positive effect (β = .073) that this predictor has via the prosocial orientation (β = .061) activated by the COVID-19 pandemic.

**Table 2:** Direct, Indirect and Total Effect That Meaning In Life (Presence and Search) has on Flourishing

Dimension	Indirect effect	Direct effect	Total effect
Presence of meaning in life			
Total	.073** [.030, .117]	.545***	.619***
Via existential orientation	.009 [-.022, .039]		
Via spiritual/religious orientation	.004 [-.012, .021]		
Via prosocial orientation	.061** [.019, .102]		
Search for meaning in life			
Total	.071** [.023, .120]	.004	.075
Via existential orientation	.008 [-.016, .037]		
Via spiritual/religious orientation	.004 [-.009, .020]		
Via prosocial orientation	.059* [.020, .105]		

*Note*. Standardized values are reported. In brackets we report the 95% confidence interval of the indirect effects.

*p < .05. **p < .01. ***p < .001.

Regarding the search for meaning in life, we found that it has a non-significant total (β = .075) as well as direct (β = .004) effect on the flourishing. Its indirect effect, instead, has a significant and positive impact (.071) on the flourishing, particularly via the prosocial orientation (β = .059).

In order to verify if the model represented in Figure 1 is invariant across believers, uncertain, and atheists, a multi-group model was run where all the paths were constrained to be the same across groups. This constrained model had very good fit indices, χ^2^(34) = 37.46, *p* = 0.313; RMSEA = .035 (95% CI [.000, .089]); *p* = 0.621; CFI = .989. Furthermore, as the freed model was a saturated model, the chi-square of this model corresponds to the Δχ^2^, which being non-significant (*p* > .05) shows that the direct, indirect and total effects of the meaning in life on the flourishing are the same regardless the emerging adult’s religious status.

### Religious Identity Model

**Figure 2:**
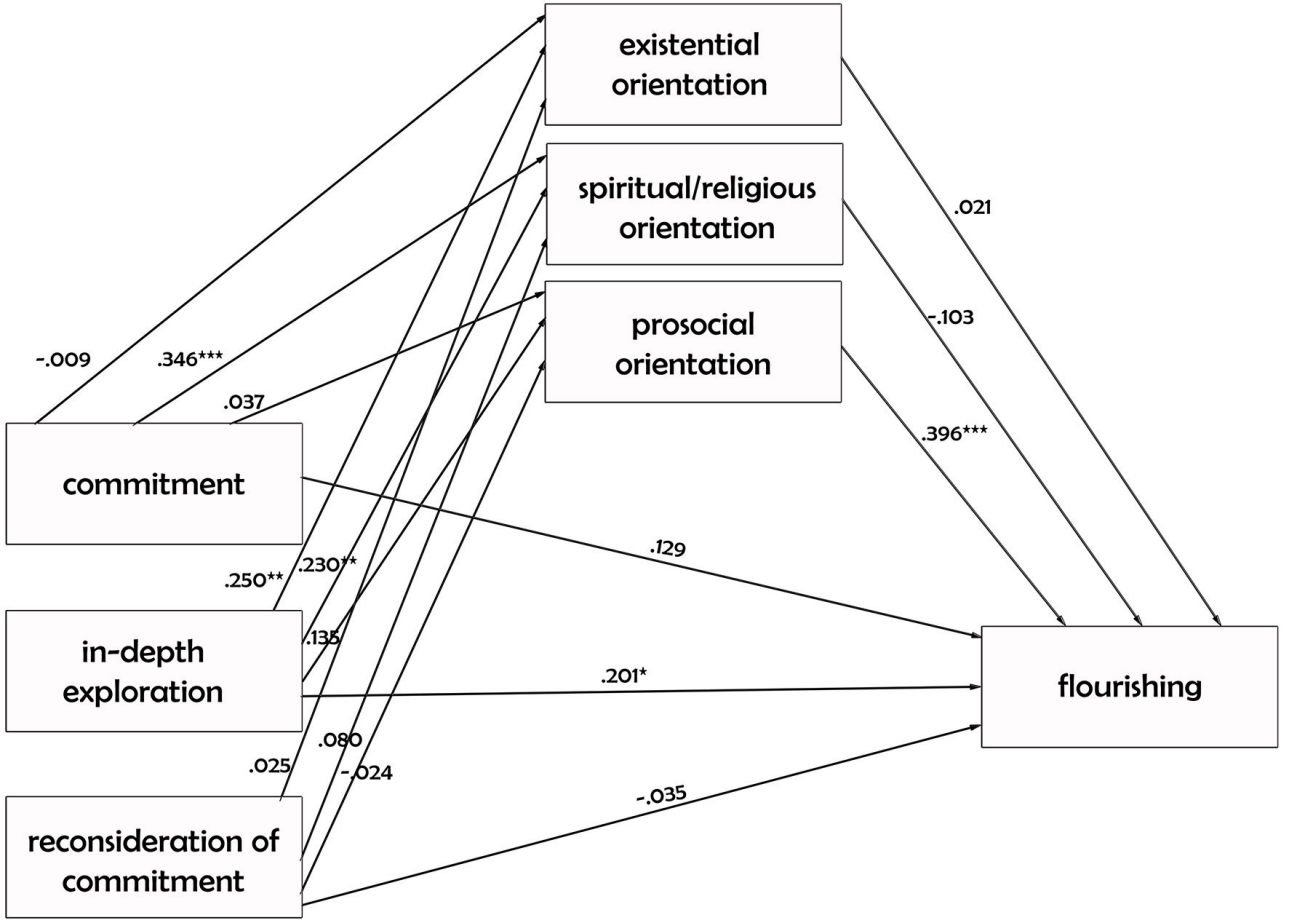
The Direct and Indirect Influence of Religious Identity On Flourishing *Note.* Saturated path analytic mediation model testing the direct and indirect influence of religious identity on flourishing (*n* = 216). For the sake of simplicity, correlations among the three predictors as well as correlations among the three mediators are not represented. Standardized values are reported. *p < .05. **p < .01. ***p < .001.

The second model testing the relationship between religious identity’s dimensions and flourishing, via three mediators was tested only on participants who filled in the religious identity scale (believer and uncertain emerging adults) (Figure 2).

Commitment is not significantly related to the flourishing, as its total (β = .108), direct (β = .129), and indirect (β = -.021) effects are not significant. Instead, the in-depth exploration has a significant total effect on the emerging adults’ flourishing (β = .236). This significant effect is attributable to the direct effect of the in-depth exploration on the outcome as it is significant (β = .201), while its indirect effect via the orientation processes is not significant (β = .035). Finally, the reconsideration of commitment is not significantly related to flourishing, as its total (β = -.052), direct (β = -.035), and indirect (β = -.017) effects are both not significant (Table 3).

**Table 3:** Direct, Indirect and Total Effect That the Religious Identity (Commitment, In-Depth Exploration, Reconsideration) Has On the Flourishing

Dimension	Indirect effect	Direct effect	Total effect
Commitment			
Total	-.021 [-.115, .073]	.129	.108
Via existential orientation	.000 [-.013, .013]		
Via spiritual/religious orientation	-.035 [-.088, .017]		
Via prosocial orientation	.015 [-.060, .089]		
In-depth exploration			
Total	.035 [-.046, .116]	.201*	.236**
Via existential orientation	.005 [-.034, .045]		
Via spiritual/religious orientation	-.024 [-.067, .019]		
Via prosocial orientation	.053 [-.017, .124]		
Reconsideration of commitment			
Total	-.017 [-.072, .037]	-.035	-.052
Via existential orientation	.001 [-.011, .012]		
Via spiritual/religious orientation	-.008 [-.027, .011]		
Via prosocial orientation	-.010 [-.063, .043]		

*Note*. Standardized values are reported. In brackets we report the 95% confidence interval of the indirect effects.

*p < .05. **p < .01. ***p < .001.

Finally, we verified if this model was invariant across believer and uncertain emerging adults. The model in which parameters were constrained to be equivalent across groups, χ^2^(22) = 23.223, *p* = 0.389; RMSEA = 0.023 (95% CI [0.000, 0.085], *p* = 0.695; CFI = 0.996] was not significantly different from the freed model (*p*Δχ^2^ > .05), indicating that the model works well both for believers and uncertain.

## Discussion

The recent threat of the COVID-19 pandemic has come with a large number of potential stressors that caused psychological distress among emerging adults worldwide (Glowacz & Schmits, 2020; Ranta et al., 2020).

Capitalizing on the idea that people, in the midst of a stressful event, may experience the potential for flourishing through the process of searching for meaning (Trzebiński et al., 2020; Yu et al.,2020), we aimed to investigate specific patterns of reflection and re-orientation in a group of emerging adults. In particular, we focused on existential issues, spirituality and religiosity, and prosociality as three primary trajectories for building meaning that may lead to flourishing.

Specifically, first we investigated the role of meaning in life on emerging adults’ flourishing during pandemic both directly and indirectly through the orientation processes. Results provided further support to previous literature about the protective role of the presence of meaning in life on psychological well-being, thus confirming that considering life as meaningful contributes to individuals’ sense of control over adverse situations such as COVID-19 emergency (Arslan et al., 2021; Karataş & Tagay, 2021; Schnell & Krampe, 2020; Trzebiński et al., 2020). Yet our findings also added a peculiar facet to the relationship between meaning and flourishing in emerging adults during pandemic. In fact, prosocial orientation emerged as a crucial mediator of the relationship between both presence and search for meaning and flourishing. This means that the process of building meaning in the midst of adversity is associated with the experience of flourishing both directly (as demonstrated in previous studies; McAdams et al., 2001) and indirectly, via prosocial behavior. The relationship between the constructs of meaning and flourishing turned out to be mediated by the motivation and capacity to engage in prosocial activities, that is those who developed a sensitivity towards others and implemented prosocial behaviors during the COVID-19 pandemic were able to activate a positive adjustment process that incremented their flourishing level. Our findings are in support of the notion that being able to engage in more prosocial behavior in the context of adversity can be considered as a protective factor against negative adjustment (Masten & Reed, 2002) and is highly connected with resilience (Russo et al., 2021). Caring about and being oriented toward others implies the tendency to transcend oneself (Ardenghi et al., 2023; Schwartz, 2012), self-actualizing and find connections with other people (Schwartz & Sortheix, 2018), which, in literature, has been recognized as a fundamental source of both meaning in life (Van Tongeren & Van Tongeren, 2021) and well-being (Sortheix & Lönnqvist, 2014). As confirmed by a recent meta-analysis (Hui et al., 2020), prosociality may significantly contribute to both the alleviation of distress and the enhancement of well-being and flourishing life (Nelson et al., 2016). Thus, cultivating meaning through developing a specific emphasis on social interactions can move people toward states of wholeness (VanderWeele, 2017). Furthermore, we found that the direct, indirect and total effects of meaning in life on flourishing are the same, regardless of the emerging adults’ religious status.

We then examined the role of religious identity—the other key aspect that people rely upon to cope with adversity (Aten et al. 2019)—on emerging adults’ flourishing during pandemic both directly and indirectly, namely through the orientations processes. Despite different studies have found that emerging adults tend to question the religious beliefs in which they were raised (Barry & Nelson, 2005) and may become less attached to the religion (McNamara Barry et al., 2010), religiosity remains a potential resource during this life stage. Results showed that only in-depth exploration has a significant direct effect on flourishing, while its indirect effect via the orientation processes was not significant. This means that actively probing one’s own religious commitment in emerging adulthood is positively associated with individual functioning. This result is partially in line with previous studies finding that religious commitment is positively associated with subjective well-being (Villani et al., 2019). Even if the lack of the effect of religious identity on prosocial orientation leading to the non-significant indirect effect on flourishing appears as inconsistent with other studies showing the association between religiosity and feelings of empathy and compassion (Markstrom et al., 2010), prosociality (Bennett & Einolf, 2017) and altruistic behaviors in young adults (Zarghi & Bolghan-Abadi, 2021), this result should be read with caution because the questionnaire used to measure religious identity does not consider the social dimension of religiosity, such as belonging to the religious community (Sorgente et al., 2022). Also in this case we found that the model works well both for believer and uncertain emerging adults keeping the COVID-19 impact variable under control. Thus, both models presented in this study are invariant across different religious statuses, although the results of the ANOVA reported some differences between groups in favor of the group of believers. This means that, despite the groups having different levels of the constructs/variables under investigation, the processes linking these constructs are the same across groups.

This study presents some limitations. First, to measure the orientation processes that people activated due to their COVID-19 we used a self-report scale which was constructed *ad hoc*, albeit on the basis of prior work. Even if we found a good reliability of the three orientation processes, there is no similar assessment tool that allows us to compare our results with those coming from other studies (Van Tongeren & Van Tongeren, 2021). Second, due to the correlational nature of the data, caution is required in the interpretation of both causal relationships and mediations among the variables. Future longitudinal designs are encouraged to better ascertain temporal ordering and causality. The third limitation is related to the need to generalize results to the national cultural context in which the relationship among variables are examined (Cohen & Johnson, 2017). Thus, as the sample was mostly composed of Italian Catholic emerging adults, we should be cautious in generalizing these results to individuals with different ages and belonging to other cultural contexts.

### Conclusion

Taken together, our results revealed a positive influence of presence of meaning and in-depth exploration on flourishing among emerging adults, thus providing further support for the potential of both meaning making and religiosity for growth and well-being in adverse times. Specifically, within this process of reflection and re-orientation, prosociality seems to be a particularly effective path to build meaning and, ultimately, to increase flourishing (VanderWeele, 2020). The link between prosociality and well-being is particularly strong among younger people who, by engaging in prosocial behaviors, not only may experience higher levels of overall well-being, but also have the chance to fulfill a crucial developmental task, that is (re)conceptualizing the self and enhancing the understanding of the purpose and meaning of their own life (Hui et al., 2020). Our findings support the idea that this process becomes even more important when emerging adults are called to face challenges that might significantly change their life trajectories and could be taken into account to sustain the emerging adults’ well-being during the pandemic. Therefore, it would be welcomed to develop interventions that directly target emerging adults and also adolescents aimed at promoting their motivation and capacity to engage in prosocial behaviors (Coulombe & Yates, 2022). Given its strong link with flourishing, promoting the development of prosocial-oriented perspectives and attitudes should become an increasing part of the educational practices offered by all the significant socialization agencies (Russo et al., 2022), especially in view of other possible challenges that the citizens of tomorrow might have to face in future.

## Supplementary Materials

The supplementary materials provided are the tables containing the factor loadings and latent correlations for the orientation scales that support the findings of this study (for access see Index of Supplementary Materials below).



VillaniD.
SorgenteA.
AntoniettiA.
IannelloP.
 (2023). Supplementary materials to "The contribution of meaning making and religiosity to individuals’ psychological wellbeing during the Covid-19 pandemic: Prosocial orientation matters"
[Tables, factor loadings, latent correlations]. PsychOpen. 10.23668/psycharchives.12872
PMC1050821037731894

## Data Availability

Data are freely available, see Villani, Sorgente, Antonietti, & Iannello (2023).
